# Safety and efficacy of Cox‐Maze procedure for atrial fibrillation during mitral valve surgery: a meta-analysis of randomized controlled trials

**DOI:** 10.1186/s13019-024-02622-0

**Published:** 2024-03-19

**Authors:** Yaxuan Gao, Hanqing Luo, Rong Yang, Wei Xie, Yi Jiang, Dongjin Wang, Hailong Cao

**Affiliations:** 1 Department of Cardio-Thoracic Surgery, Nanjing Drum Tower Hospital, The Affiliated Hospital of Nanjing University Medical School, Nanjing, 210008 Jiangsu China; 2 Institute of Cardiothoracic Vascular Disease, Nanjing University, Nanjing, Jiangsu China; 3 Nanjing Drum Tower Hospital Clinical College of Nanjing University of Chinese Medicine, Nanjing, Jiangsu China

**Keywords:** Atrial fibrillation, Mitral valve surgery, Cox-Maze procedure, Meta-analysis

## Abstract

**Background:**

Cox‐Maze procedure is currently the gold standard treatment for atrial fibrillation (AF). However, data on the effectiveness of the Cox‐Maze procedure after concomitant mitral valve surgery (MVS) are not well established. The aim of this study was to assess the safety and efficacy of Cox-Maze procedure versus no-maze procedure n in AF patients undergoing mitral valve surgery through a systematic review of the literature and meta‐analysis.

**Methods:**

A systematic search on PubMed/MEDLINE, EMBASE, and Cochrane Central Register of Clinical Trials (Cochrane Library, Issue 02, 2017) databases were performed using three databases from their inception to March 2023, identifying all relevant randomized controlled trials (RCTs) comparing Cox-Maze procedure versus no procedure in AF patients undergoing mitral valve surgery. Data were extracted and analyzed according to predefined clinical endpoints.

**Results:**

Nine RCTs meeting the inclusion criteria were included in this systematic review with 663 patients in total (341 concomitant Cox‐Maze with MVS and 322 MVS alone). Across all studies with included AF patients undergoing MV surgery, the concomitant Cox‐Maze procedure was associated with significantly higher sinus rhythm rate at discharge, 6 months, and 12 months follow‐up when compared with the no-Maze group. Results indicated that there was no significant difference between the Cox‐Maze and no-Maze groups in terms of 1 year all-cause mortality, pacemaker implantation, stroke, and thromboembolism.

**Conclusions:**

Our systematic review suggested that RCTs have demonstrated the addition of the Cox‐Maze procedure for AF leads to a significantly higher rate of sinus rhythm in mitral valve surgical patients, with no increase in the rates of mortality, pacemaker implantation, stroke, and thromboembolism.

**Supplementary Information:**

The online version contains supplementary material available at 10.1186/s13019-024-02622-0.

## Introduction

Atrial fibrillation, which is associated with a doubling of cardiovascular mortality and increased risk of stroke and systemic emboli, is present in 30 to 50% of patients presenting for mitral-valve surgery [1]. Therapeutic strategies (pharmacological, catheter ablation, antiarrhythmic surgery) for atrial fibrillation aim to diminish uncomfortable symptoms such as tachycardias and palpitations, by restoring sinus rhythm, improving hemodynamics, reinstituting atrioventricular synchrony and reducing thromboembolic risk by reproducing bi-atrial contraction [2, 3]. The development of open surgical procedures for the Cox-Maze procedure of atrial fibrillation has led to their widespread application during cardiac operations [4–6], but their effectiveness and safety have not been rigorously established.

The Cox-Maze procedure (CMP) is one of the most effective surgical treatments for atrial fibrillation (AF) [7]. The MAZE operation, as an open-heart surgical procedure, was introduced by James L. Cox in 1987 as a ‘cut-and-suture’ technique, a technique based on a multiple wavelet theory, which proposes that different depolarizing wavefronts circle the atria [8]. MAZE incisions should reduce the atrial mass below the critical reentry circuit size, preventing atrial fibrillation [7]. The Cox‐Maze IV is currently the gold standard surgical treatment for AF, with estimated freedom from AF and from antiarrhythmic drugs at 1 year postoperatively of 93% and 85%, respectively [9].

The 2012 Heart Rhythm Society/European Heart Rhythm Society/European Cardiac Arrhythmia Society guidelines recommend AF intervention as an acceptable treatment in symptomatic patients, concomitant to other cardiac surgery (level C evidence) [10]. Despite this, many patients with AF undergoing cardiac surgery for other pathologies are not offered a concomitant CMP, whereas other patients with lone AF are not offered surgical intervention at all [11]. Gabriella Boano demonstrated that the current data cannot exclude that the addition of the Cox-Maze IV procedure to MVS increases the risk of PHF (postoperative heart failure), possibly by increasing the cross-clamp time [12]. Moreover, Cox-Maze procedure was associated with an increased risk for permanent pacing, and PPM implantation following MVS was associated with a significant increase in 1 year mortality [13]. It has been estimated that approximately one-third of patients undergoing mitral valve surgery who have a history of AF do not receive the CMP [14].

Previous meta-analyses and systematic reviews have assessed diverse populations undergoing multiple types of ablation surgery, including pulmonary vein isolation (PVI), as well as catheter ablation [15, 16]. As such, there are high levels of heterogeneity in some of the outcomes reported, likely derived from such mixed surgical populations. The efficacy of Cox-Maze procedure in patient populations undergoing only mitral valve surgery is not well established. For this purpose, the present meta-analysis aims to provide randomized evidence to evaluate the safety and efficacy of Cox-Maze in AF patients undergoing mitral valve surgery.

## Methods

### Study design

The Preferred Reporting Items for Systematic Reviews and Meta-Analyses (PRISMA) 2020 Statement was followed in this systematic review and meta-analysis study. Because this study was a systematic review and meta-analysis, patient-informed consent and ethical approval were not required.

### Literature search strategy

This systematic review was registered and accepted for inclusion in PROSPERO in July 2023 (PROSPERO ID Number: CRD42023441043). We searched PubMed, Embase, and Cochrane Central Register of Clinical Trials (Cochrane Library, Issue 02, 2017) databases from 1987 up to 15th of March 2022. We used the following terms: (‘atrial fibrillation’ OR AF OR dabigatran OR rivaroxaban OR apixaban) AND (ablation OR ‘catheter ablation’). The reference lists of identified articles were also reviewed for additional sources.

### Study selection and outcomes

After deduplication, study eligibility was assessed independently by two investigators reviewing each retrieved article (H Luo and R Yang). Any discrepancies were resolved by discussion and consensus between the three investigators (H Luo, R Yang and Y Gao). The final results were reviewed by the senior investigator (H Cao). The studies were selected through the following two levels of screening: in the first step studies were independently screened based on titles and abstracts, and in the second step, full‐text reports were evaluated based on predefined criteria. Studies were eligible in patient cohorts who underwent mitral valve surgery concomitantly with the treatment of AF, which utilized Cox-Maze application, including Cox-Maze III (cut and sew), Cox-Maze (IV), or individualized modified Cox-Maze procedures (radiofrequency ablation, cryoablation, and microwave ablation), and met the following inclusion criteria:

1. Population: adults or adolescents (12 years or older);
2. Comparator: Cox-Maze procedures versus no-maze procedures in AF patients undergoing mitral valve surgery
3. Provided outcomes: death, sinus rhythm at 12‐months follow‐up;
4. Design: RCT with at least 10 patients per treatment of interest;
5. Published in English language and to those involving human subjects, Abstracts, case reports, conference presentations, editorials, and expert opinions were excluded.

Studies were ineligible if they had follow‐up shorter than 12 months and if they were duplicates. For studies reported in more than one publication, or when institutions reported subsequent studies with accumulating numbers of patients or increased lengths of follow‐up, only the most complete reports (in terms of reported outcomes and control of confounding) were included.

The primary outcomes were recurrence of AF and mortality after a 12‐month follow‐up. The secondary outcomes included aortic cross‐clamp time (XCT), CBP time, rate of MV repair, and duration of preoperative AF.

### Data extraction and critical appraisal

We assessed and extracted data on study characteristics, patients' baseline data, and data regarding study outcomes independently by two investigators (H Luo and R Yang) with verification for accuracy by two other investigators (Y Gao and W Xie). The investigators looked for information on the sources of funding for individual studies included in the review, but it was not required to be reported. Digitizing software was used to recover graphically presented data. Where necessary, study authors were contacted to obtain additional information.

### Risk of bias-study quality assessment

Risk of bias in individual studies was assessed independently by two investigators. Discrepancies were resolved through discussion between three investigators. RCTs were assessed using the revised Cochrane Collaboration Risk of Bias tool 2 (RoB2) for RCTs. The domains included in RoB 2 cover all types of bias that are currently understood to affect the results of randomized trials. These are 1. Bias arising from the randomization process; 2. Bias due to deviations from intended interventions; 3. Bias due to missing outcome data; 4. Bias in measurement of the outcome; and 5. Bias in selection of the reported result.

### Statistical analysis

Descriptive statistics are presented as the number of cases (n) for dichotomous and categorical variables. Statistical analysis was performed in line with recommendations from the Cochrane Collaboration and PRISMA guidelines, using STATA software (version 16.0, StataCorp, College Station, Texas, USA, the Cochrane Collaboration, 2014). Heterogeneity was assessed using the I^2^ statistics, which is the proportion of total variation observed among the studies attributable to differences between studies rather than sampling error (chance). Data were summarized across groups using the Mantel- Haenszel Fixed-Effect model if I^2^ < 25. We considered I^2^ less than 25% as low and I^2^ greater than 75% as high. The random effects model was used if I^2^ > 25%. If there was substantial heterogeneity, the possible clinical and methodological reasons for this were explored qualitatively. In the present meta-analysis, the results using the random-effects model were presented to take into account the possible clinical diversity and methodological variation between studies. We used methods adapted from TSA (trial sequential analysis) applied to cumulative meta-analysis to assess the reliability and conclusiveness of the available evidence on the primary outcome, focusing on the risk of 1 year all-cause mortality. The sample size needed for a reliable and conclusive meta-analysis is at least as large as that for a single optimally powered randomized controlled trial, so we calculated the sample size (optimal information size) requirement for our meta-analysis. We did TSA for meta-analyses by using the optimal information size to help construct a boundary for our meta-analysis. We used this boundary as a way of determining whether the evidence in our meta-analysis was reliable and conclusive. All P values were 2-sided.

## Results

**Fig. 1:**
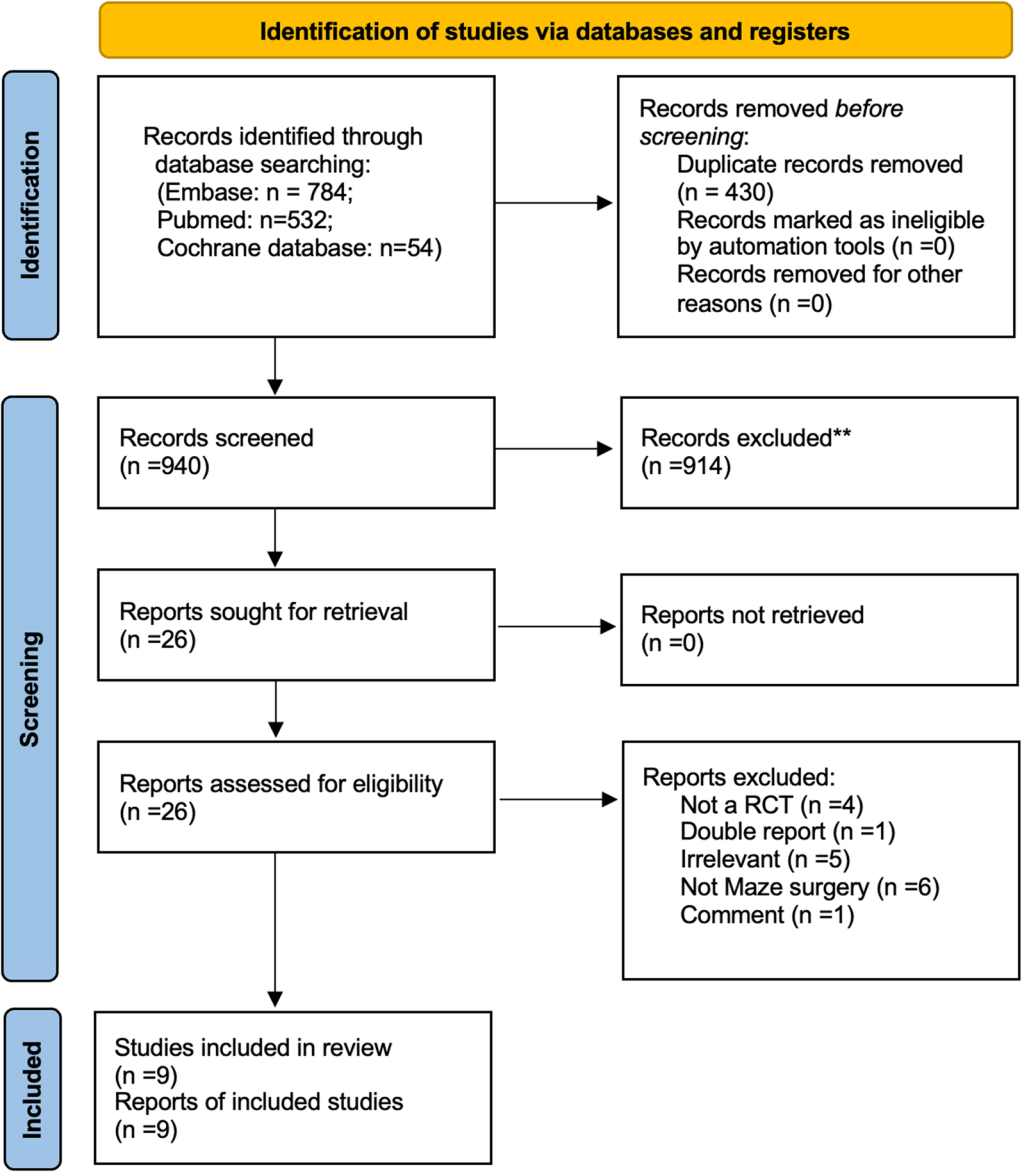
PRISMA (Preferred reporting items for systematic reviews and meta‐analyses) flow diagram. RCT, randomized controlled trial

A total of 1370 studies were identified from the literature search, after 430 duplicate records were removed, 940 potentially relevant articles were retrieved. After a detailed evaluation of these articles, 913 were deemed irrelevant to the research question, and 26 required reviews of the full‐text article. After screening, 17 studies did not match the study criteria. All of the included studies were RCTs (Level 1 evidence) [17–25]. Overall, nine RCT clinical studies involving 663 patients treated with (MVS + MAZE group; n = 341) or without MAZE procedures (MVS group; n = 322) for atrial fibrillation after MVS met the inclusion criteria and were included in the meta‐analysis. Five studies used Cox-Maze IV (radiofrequency ablation) [17, 21–24], one study used Cox-Maze IV (radiofrequency ablation) with port-access [18], one study used traditional Cox-Maze III (cut and sew) [19], one study used Modified Cox-Maze III (cut and sew + electrocoagulation) [20], one study used Modified Cox-Maze III (cut and sew + cryoablation) [25]. All nine study patients underwent treatment of the left atrial appendage, depending on the surgeon's choice and the specific condition of the patient's left atrial appendage, Specific surgical procedures including closure of the left atrial appendage, excising left atrial appendage, radio-frequency ablation. Figure 1 shows the full PRISMA flow diagram with information about the selected, included, and excluded clinical studies.

### Quality of studies

**Fig. 2:**
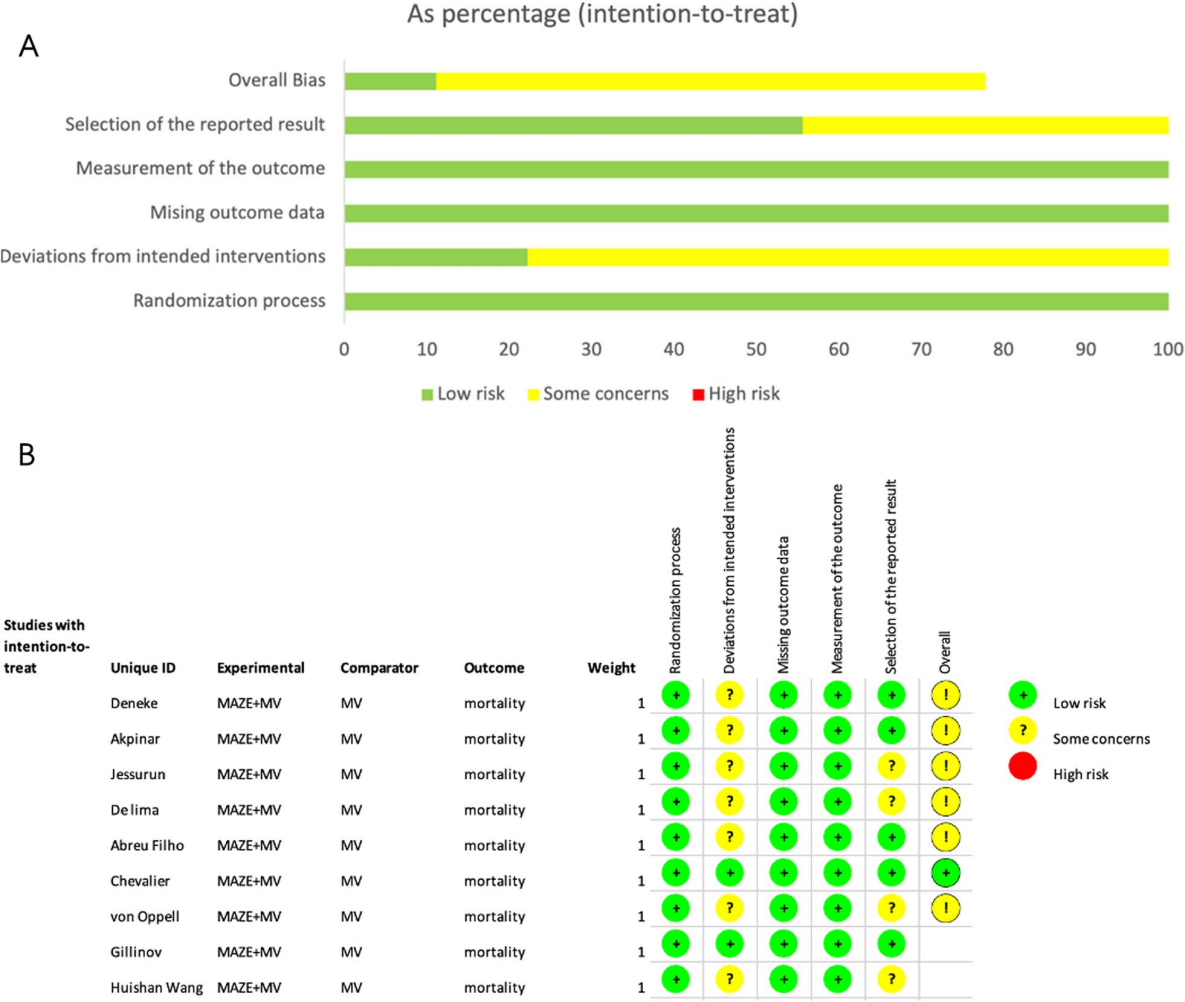
**A** Risk of bias graph. Risk of bias graph: review authors’ judgements about each risk of bias item presented as percentages across all included studies. **B** Risk of bias summary

The 9 RCTs were also assessed qualitatively using tools recommended by the Cochrane Collaboration Risk of Bias tool 2 (RoB2) for RCTs, A graph and summary of 5 domains included in RoB 2 cover all types of bias identified in each individual RCT is shown in Fig. 2.

### Study characteristics

The year of publication ranged from 2002 to 2018. Details of individual studies and patients' baseline characteristics including the history of AF, intraoperative data including antiarrhythmic drugs, and mid‐term postoperative outcomes are summarized in Tabled 1, 2, and 3, Additional file 2: Supplementary Appendix. The AF therapies before surgery are summarized in Additional file 1: Supplementary Appendix. Briefly, 5 (55.6%) studies were implemented in the population in Europe, 2 (22.2%) studies were in South America, 1 (11.1%) was in North America, and 1 (11.1%) in Asia (Table 1). With regard to study design, all 9 studies were RCTs (Level 1 evidence), and similar baseline characteristics were observed in both comparison arms.

**Table 1:** Summary of RCTs comparing MV + COX versus MV only surgical treatment in AF patients

First author	Year	Country	Study period	MVS + MAZE	MVS	Primary endpoint
Deneke, T	2002	Germany	1998–1999	15	15	SR
Akpinar, B	2003	Turkey	NR	33	34	AF free
Jessurun, E. R	2003	Netherlands	1996–1999	25	10	SR
de Lima, G. G	2004	Brazil	1999–2001	10	10	SR
Abreu Filho, C. A	2005	Brazil	2000–2002	15	14	AF free
Chevalier, P	2009	France	2002–2005	21	22	SR
von Oppell, U. O	2009	United Kingdom	2004–2006	24	25	SR
Gillinov, A. M	2015	USA	2010–2013	133	127	AF free
Wang, H	2018	China	2013–2015	65	65	Freedom from stroke or death

**Table 2:** Summary of baseline patient characteristics in Maze + MVS with MVS alone in surgical treatment for AF

First author	Age		Male (%)		LVEF (%)		LAD (mm)		AF duration (mo)		Mitral stenosis(%)		Mitral regurgitation(%)		Permanent or persistent AF	
	Maze + MVS	MVS	Maze + MVS	MVS	Maze + MVS	MVS	Maze + MVS	MVS	Maze + MVS	MVS	Maze + MVS	MVS	Maze + MVS	MVS	Maze + MVS	MVS
Deneke, T	64.7	69.7	40.0	20.0	64.0	61.0	59.8	57.8	43.2	44.4	13.3	20.0	53.3	66.7	100.0	100.0
Akpinar, B	53 ± 10	50 ± 8	60.1	73.5	55.2 ± 6.3	55.0 ± 8.1	62.5 ± 10.5	66.7 ± 9.0	19.9 ± 10.6	22.0 ± 13.9	NR	NR	NR	NR	NR	NR
Jessurun, E. R	64 ± 12	64 ± 9	56.0	50.0	45.0 ± 15.0	45.0 ± 6.0	53.0 ± 9.0	56.0 ± 7.0	NR	NR	NR	NR	NR	NR	48.0	80.0
de Lima, G. G	50.1 ± 15.3	50.1 ± 15.4	30.0	60.0	64.3 ± 7.0	64.0 ± 9.5	60.0 ± 16.0	62.0 ± 12.0	14.0	16.5	40.0	20.0	50.0	50.0	100.0	100.0
Abreu Filho, C. A	55.4 ± 12.8	50.7 ± 9.7	33.3	42.9	62.8 ± 9.2	66.1 ± 10.5	61.1 ± 11.2	58.8 ± 4.7	66.1 ± 57.4	43.8 ± 28.5	61.9	64.2	38.1	35.8	100.0	100.0
Chevalier, P	69.1 ± 6.2	66.3 ± 9.7	23.8	50.0	59.8 ± 8.5	61.3 ± 9.5	54.6 ± 10.9	52.6 ± 11.0	161.0	89.0	19.1	22.7	57.1	63.6	100.0	100.0
von Oppell, U. O	66 ± 8	68 ± 9	33.0	56.0	52 ± 12	55 ± 16	52 ± 8	55 ± 8	84 ± 120	60 ± 48	8.0	0.0	63.0	60.0	91.7	88.0
Gillinov, A. M	69.7 ± 10.4	69.4 ± 10.0	57.1	50.4	55.1 ± 7.6	56.5 ± 7.7	NR	NR	NR	NR	NR	NR	43.6	42.5	42.1	49.2
Wang, H	55.92 ± 7.17	55.37 ± 7.67	29.2	41.0	55 ± 4	54 ± 3	51.8 ± 8.0	51.4 ± 8.4	NR	NR	95.3	93.8	76.8	80.0	100.0	100.0

**Table 3:** Summary of perioperative characteristics

First author	CBP time (min)		Cross-clamp time (min)		MVP (%)		MVR (%)		Other surgeries		Mortality at 30 d		Mortality at 12mo		Thromboembolic at12mo	
	Maze + MVS	MVS	Maze + MVS	MVS	Maze + MVS	MVS	Maze + MVS	MVS	Maze + MVS	MVS	Maze + MVS	MVS	Maze + MVS	MVS	Maze + MVS	MVS
Deneke, T	188.0	127.0	103.0	85.0	0.0	0.0	100.0	100.0	0.0	0.0	13.0	0.0	73.0	93.0	0.0	0.0
Akpinar, B	140.5 ± 34.3	128.3 ± 28.3	88.5 ± 13.4	78.9 ± 9.4	36.0	38.0	64.0	62.0	27.3	20.6	3.0	2.9	3.0	2.9	0.0	6.0
Jessurun, E. R	155.0 ± 27.0	97.0 ± 27.0	90.0 ± 24.0	60.0 ± 18.0	40.0	40.0	60.0	60.0	24.0	30.0	0.0	0.0	0.0	0.0	0.0	0.0
de Lima, G. G	115.3 ± 25.0	68.3 ± 22.0	NR	49.1 ± 19.0	80.0	60.0	20.0	40.0	0.0	0.0	0.0	0.0	10.0	0.0	0.0	10.0
Abreu Filho, C. A	107.2 ± 21.1	78.2 ± 24.4	67.5 ± 13.5	47.1 ± 15.8	30.9	3.5	69.1	96.5	26.1	28.5	2.3	0.0	4.9	7.2	0.0	0.0
Chevalier, P	NR	NR	93 ± 32	74 ± 19	19.0	59.9	45.5	80.9	19.0	40.9	4.8	0.0	4.8	0.0	4.8	13.6
von Oppell, U. O	176 ± 42	160 ± 55	143 ± 35	119 ± 44	66.7	60.0	33.3	40.0	54.1	64.0	NR	NR	0.0	8.0	4.0	0.0
Gillinov, A. M	147.8 ± 63.3	132.5 ± 51.0	102.9 ± 41.5	95.9 ± 36.3	59.4	51.6	40.6	48.4	37.6	38.1	NR	NR	6.8	8.7	3.0	1.6
Wang, H	164.82 ± 30.57	107.03 ± 18.96	88.06 ± 13.46	54.20 ± 8.49	0.0	0.0	100.0	100.0	33.8	32.3	1.5	1.5	6.2	13.9	1.5	4.6

Other main features of clinical studies are reported in Tables 2, 3. Of note, demographic characteristics (including age and sex) were available in 218 patients (96%). Left ventricular ejection fraction (LVEF) was available in 183 patients (81%) from eight studies and left atrial anteroposterior diameter was available in 156 patients (69%). The mitral stenosis rate and mitral regurgitation rate are demonstrated in Table 2. Permanent AF, persistent AF, and a mixture of permanent and persistent AF populations were evaluated by four studies [17, 20–22], three studies [18, 24, 25], and two studies [19, 23], respectively.

All patients underwent MVS. Briefly, 61.6% and 38.4% of patients underwent MV replacements with biological or mechanical prostheses and mitral valve repair, respectively. All included studies reported concomitant Cox‐Maze III or IV procedures performed in a standardized or modified fashion. CBP and Cross-clamp time across the studies are demonstrated in Table 3. CBP time was significantly longer when MV surgery was performed concomitantly with Cox-Maze. With the exception of one study [22] which did not report CBP, all other studies reported CBP times that demonstrated significantly longer average CBP for the MV + SA group (weighted mean: 152.1 vs. 122.7 min; *P* = 0.024). Similarly, Except for one study that did not report cross-clamp time [20], cross-clamp time was longer for the Cox-Maze + MVS group compared to MVS (weighted mean: 76.4 vs. 63.8 min; *P* = 0.096).

### Assessment of safety

**Fig. 3:**
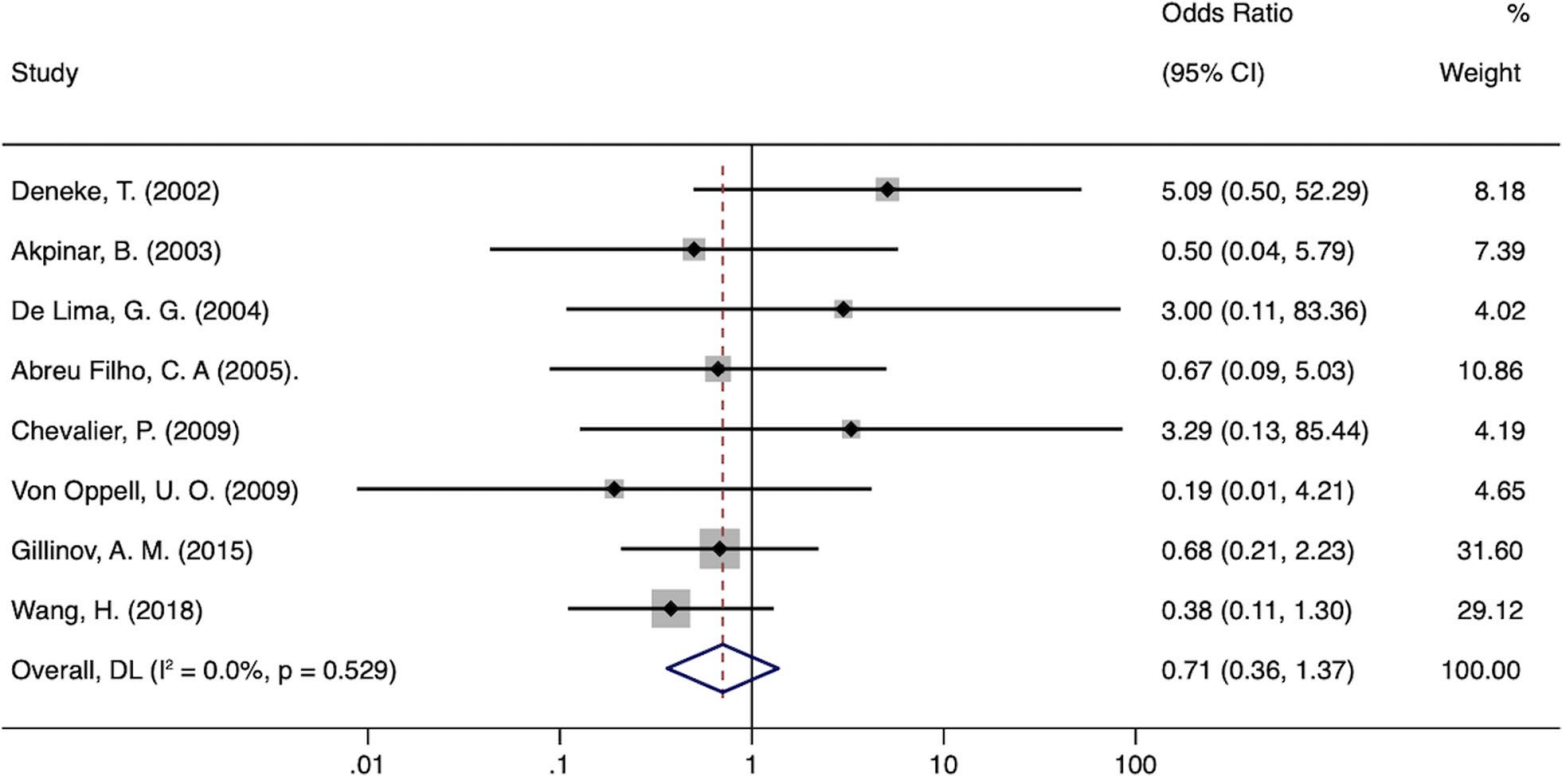
Forest plot of the odds ratio (OR) of all-cause mortality in AF patients with surgical ablation (SA + MV) or without ablation (MV)

**Fig. 4:**
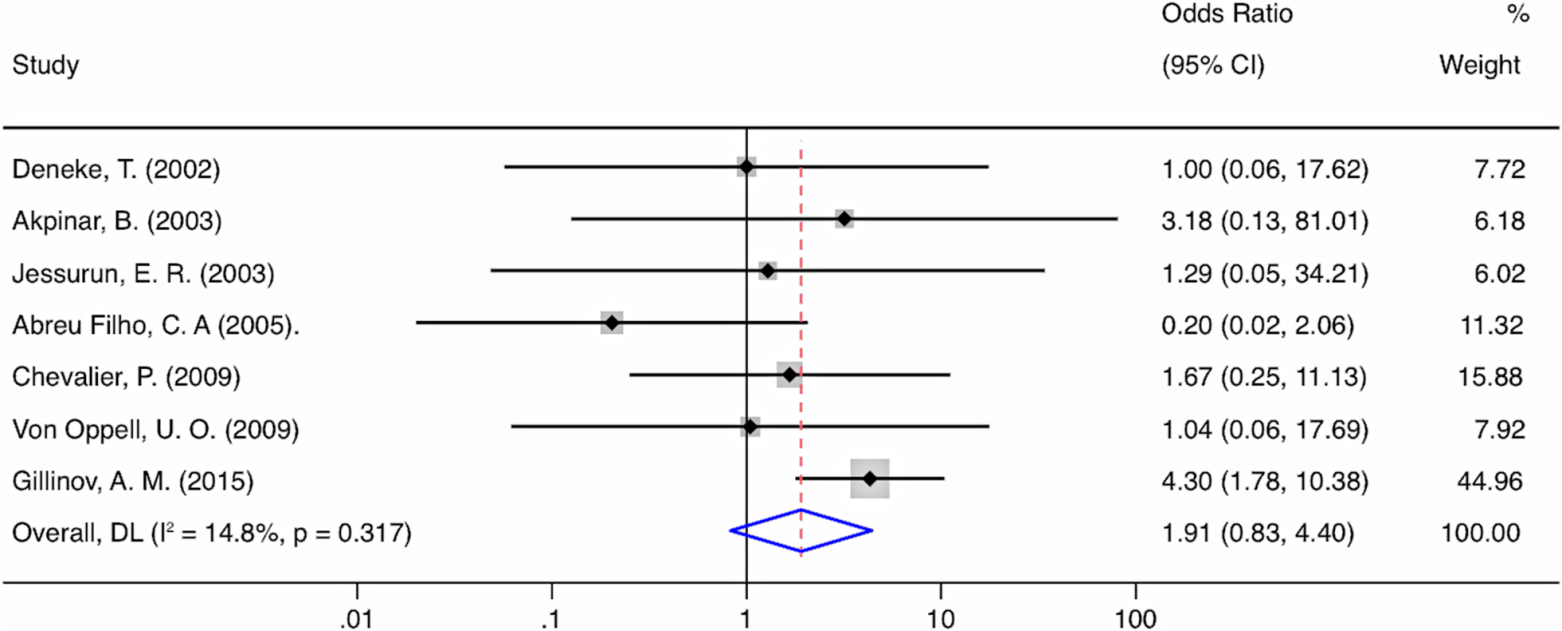
Forest plot of the odds ratio (OR) of permanent pacemaker implantation in AF patients with surgical ablation (MV + SA) or without ablation (MV)

**Fig. 5:**
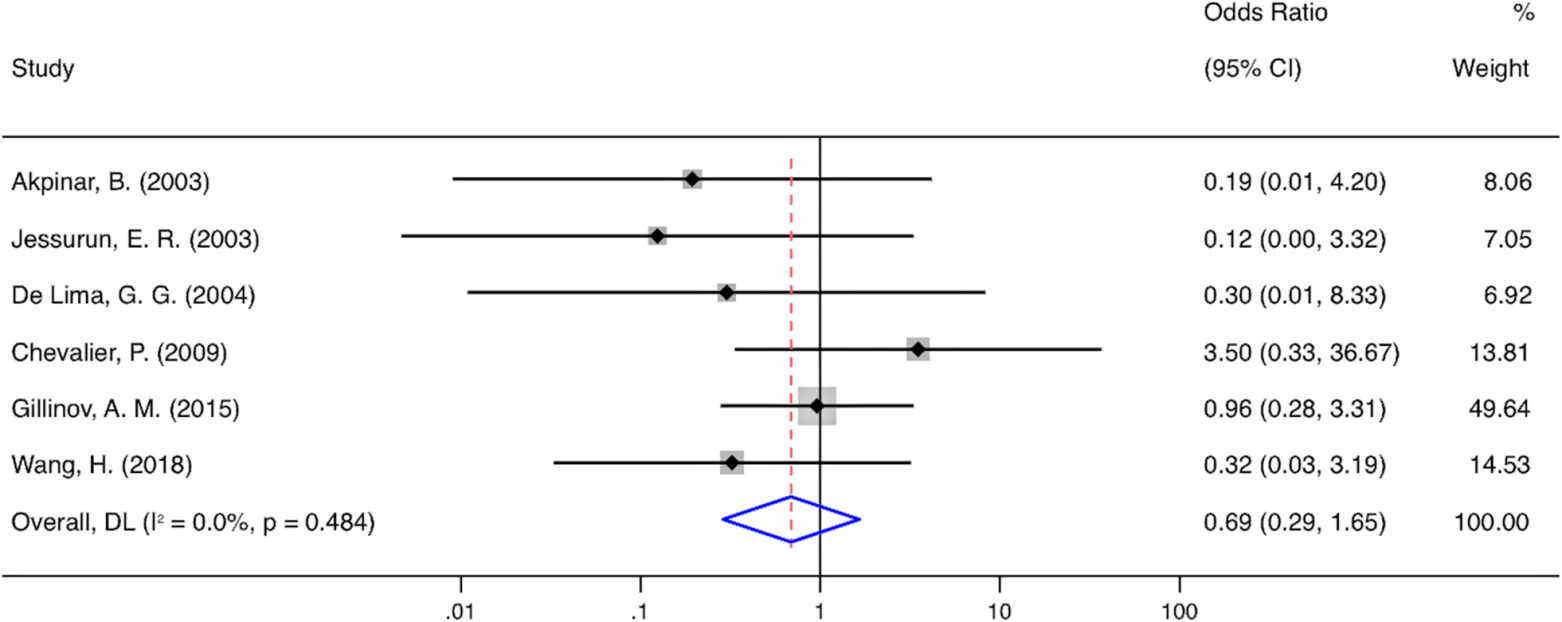
Forest plot of the odds ratio (OR) of stroke and thromboembolic events in AF patients with surgical ablation (MV + SA) or without ablation (MV)

The risk of 1 year all-cause mortality was not significantly different between MVS + Maze and MVS groups (OR 0.71; 95% confidence interval (CI) 0.36–1.37; *P* = 0.304; *I*^2^ = 0%; Fig. 3). No significant heterogeneity was observed in these two comparisons. All but three studies reported outcomes for thromboembolic events [17, 21, 23]. There were no significant differences between groups regarding thromboembolic or stroke events. The thromboembolic or stroke rates in the MVS + Maze and MVS groups were 0.6% and 0.8%, respectively (OR 0.69, 95% CI 0.29–1.65; *P* = 0.401; *I*^2^ = 0%; Fig. 4). All but two studies reported outcomes for pacemaker implantation [20, 25]. Overall, there was no difference in pacemaker implantations whether Maze was performed or not (OR 1.91; 95% CI 0.83–4.40; *P* = 0.128; I^2^ = 0%; Fig. 5).

### Persistence of sinus rhythm (SR)

**Fig. 6:**
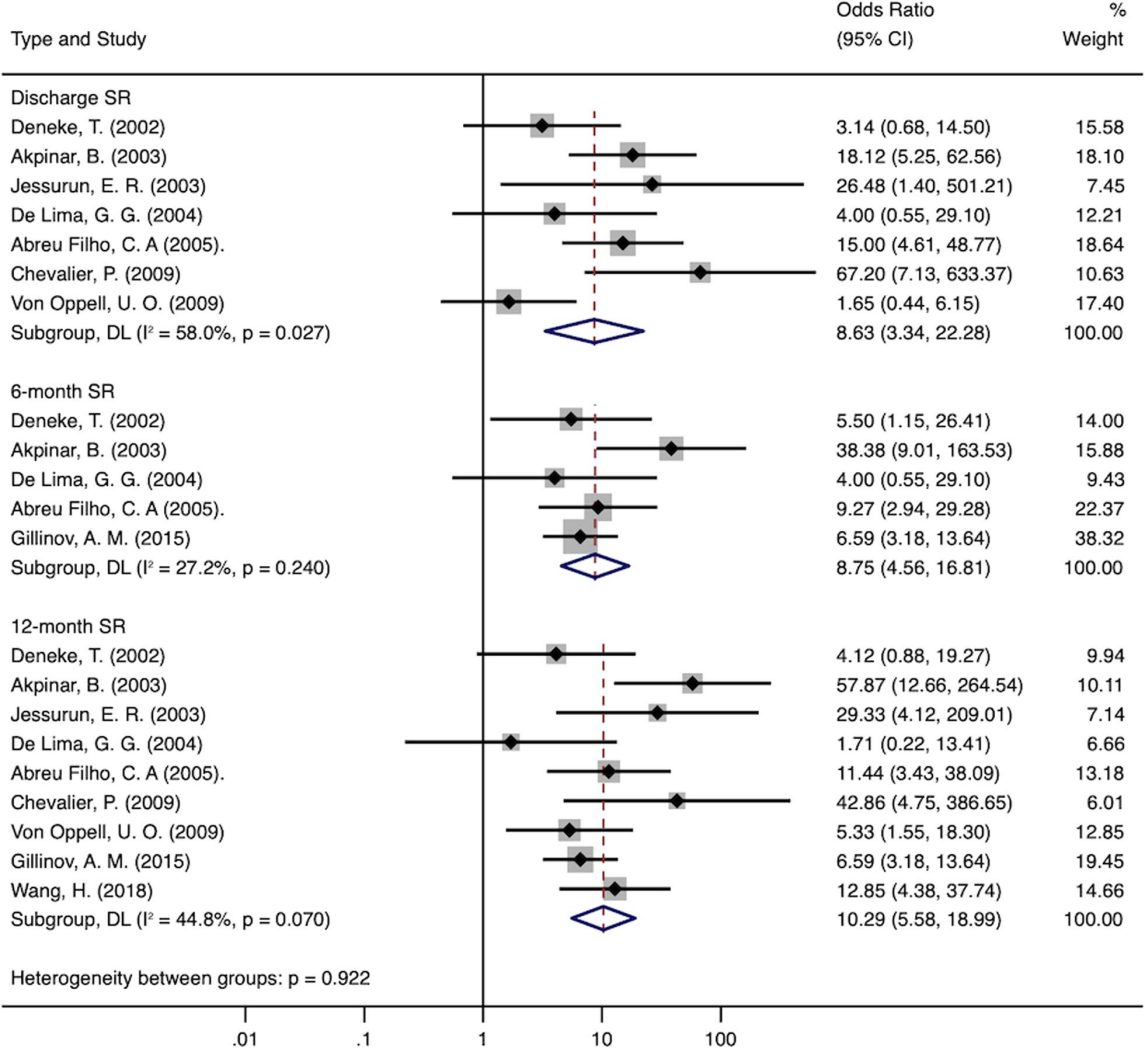
Forest plot of the odds ratio (OR) of discharge, 6 months, 12 months and > 1 year SR in AF patients with surgical ablation (MV + SA) or without ablation (MV)

Low heterogeneity was noted across clinical studies with SR follow-up results. The number of patients in SR at discharge was significantly higher in the MVS + Maze group compared to the MVS group (OR 8.63; 95% CI 3.34–22.28; *P* < 0.00001; I^2^ = 58%). At the 6 months follow-up, (OR 8.75; 95% CI 4.56–16.81;* P* < 0.00001; I^2^ = 27.2%). At the 12 months follow-up, (OR 10.29; 95% CI 5.58–18.99; *P* < 0.00001; I^2^ = 44.8%). The results are summarized in Fig. 6. However, CI (from 3.34 to 22.28 fold higher risk than control) and prediction intervals around the pooled estimates were wide (Fig. 2B) illustrating uncertainty when comparing discharge SR among patients who underwent MVS + Maze and MVS alone.

### Reliability and conclusiveness of composite outcome result

**Fig. 7:**
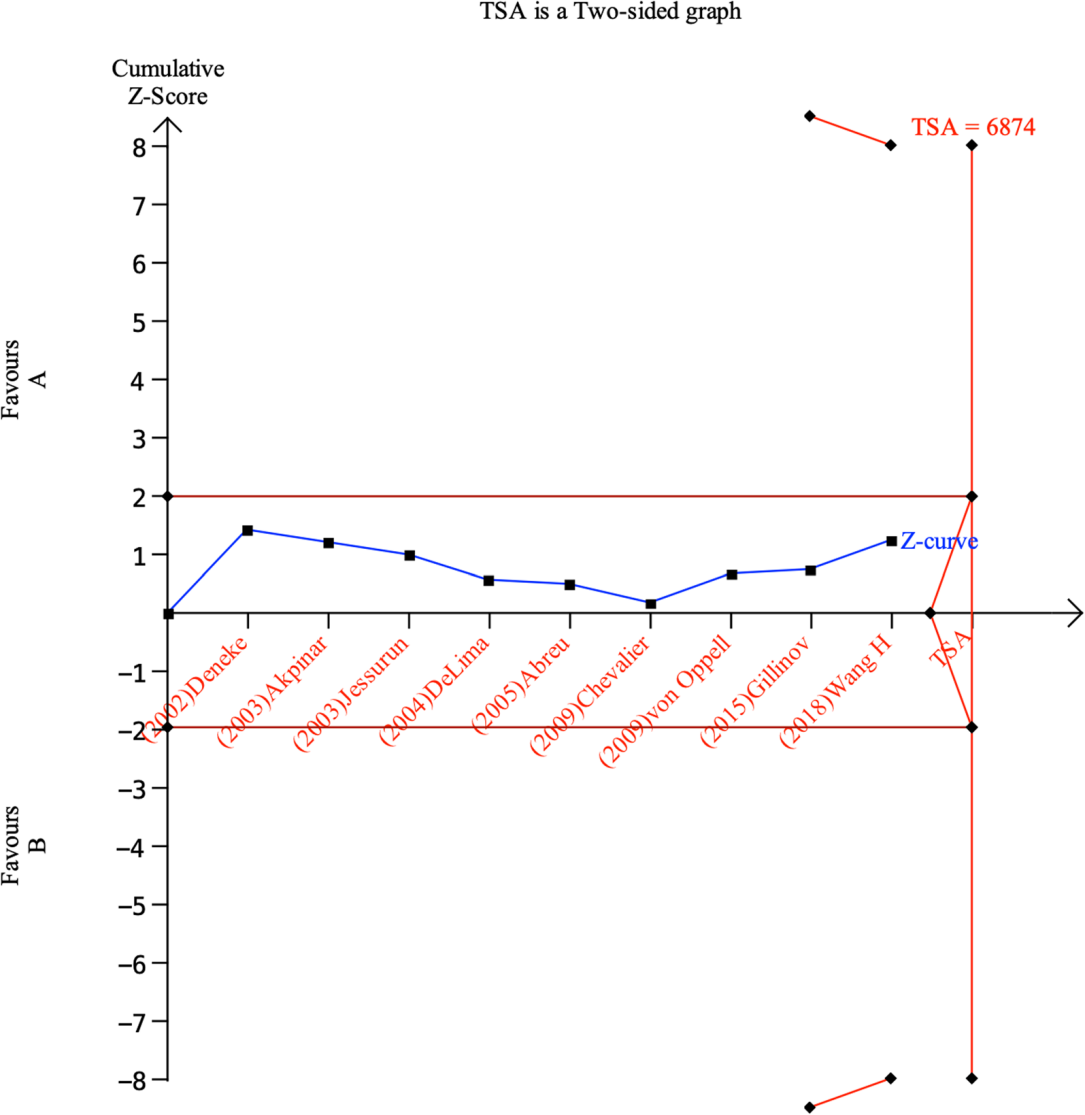
Cumulative meta-analysis assessing the effect of Cox-Maze procedures on the risk of 1 year all-cause mortality in AF patients having mitral valve surgery. The TSA boundary, which assumes a 10% control event rate and a 25% relative risk reduction with 80% power and a two-sided = 0.01, has not been crossed, indicating that the cumulative evidence is inconclusive

To determine the optimal information size we assumed a 10% control event rate (the control event rate in our meta-analysis for the composite outcome) and a 25% relative risk reduction with 80% power and a 0.01 two-sided. Our calculations indicated that the optimal information size needed to reliably detect a plausible treatment effect, for the composite outcome of **t**he risk of 1 year all-cause mortality, is 6874 patients. We used the optimal information size to help construct the TSA (trial sequential analysis) (Fig. 7). The sequential boundary has not been crossed, indicating that the cumulative evidence is unreliable and inconclusive.

## Discussion

Due to the pathomechanism of mitral valve disease, which includes left atrial enlargement due to constant pressure and volume overload, the prevalence of atrial fibrillation is high [26]. The occurrence of AF worsens the hemodynamic tolerance of MS and markedly increases the risk of thromboembolic events [27]. The weight of this evidence has provided the impetus for the combination of surgical AF treatment and mitral valve surgical intervention, with the hope of synergistic improvements in both SR prevalence and risk of morbidity and mortality. A previous meta-analysis that pooled the clinical outcomes of MV alone versus MV + various types of radiofrequency ablation of atrial fibrillation included 4 radiofrequency studies and 3 Cox-Maze studies, as well as 1 cryoablation study, and 1 pulmonary vein isolation study; nevertheless, there is still a lack of research trials or large registries evaluating surgical Cox-Maze techniques according to their various subgroups [28]. The most effective surgical option for the management of AF has been the Cox-Maze procedure (CMP), introduced by James Cox and colleagues in 1987 [7]. The outcomes of concomitant Cox‐Maze surgery during MV surgery have been extensively evaluated, but comparative data of Cox‐Maze surgery and no-maze during MVS on the mortality and freedom from AF are still limited.

The aim of any atrial fibrillation surgery is to diminish uncomfortable symptoms of the arrhythmia by restoring sinus rhythm, reinstituting atrioventricular synchrony, and reducing the risk of thromboembolic complications [29]. Restoration of atrial transport function to improve hemodynamics is another important goal of the Maze procedure. Poor left atrial contractility can cause a decrease in cardiac output of as much as 30% [30].Atrial transport function can be restored in 70% to 100% of patients after the Maze procedure. In this systematic review, we investigated the comparison of mid‐term clinical outcomes between MV + Cox-Maze versus MV alone.

Given that AF has consistently been shown to be an independent predictor of mortality [31, 32], the maintenance of SR is vital for quality of life and survival. Khiabani’s survival analysis has previously shown a survival benefit to performing concomitant AF Cox-Maze procedure as opposed to not treating the AF but this also suggests a further benefit to successful restoration of sinus rhythm [33, 34], over and above that seen with just managing the left atrial appendage (LAA). As such, the prevalence of SR at both short- and long-term follow-up is the most important endpoint considered for AF patients. Although selected single-center studies have shown rates of post-ablation freedom from atrial fibrillation of 80% or more, 1 year estimates of approximately 70% are more typical. As compared with long-term monitoring, spot ECG recordings tend to overestimate success by approximately 12 percentage points. Our results demonstrate significant improvement in the restoration of SR in the Cox-Maze group compared to the control group at discharge (66.5% vs. 18.7%), 6 months (75.5% vs. 24.2%), and 12 months (67.1% vs. 21.4%) follow-up periods. These results are consistent with previous meta-analyses involving mixed surgical populations.

The major goal of the Cox-Maze Procedure is the termination of AF and restoration of normal sinus rhythm. A secondary goal is the excision or exclusion of the left atrial appendage (LAA) to prevent strokes, the most dreaded complication of AF [7]. In previous studies, surgical ablation using the Cox-Maze technique in AF patients undergoing concomitant surgery was demonstrated to have a potential protective effect from stroke and thromboembolism in the long term. One of the most likely reasons for this may be the cessation of stagnant blood flow in the fibrillating left atrium (LA) that serves as a nidus for thromboembolic. The results of the present meta-analysis also indicate no significant difference in the incidence of stroke and thromboembolic events in favor of the surgical Cox-Maze group compared with the control MV surgery group (mean: 3.1% vs. 5.2%; *P* = 0.40). Ablation was associated with a significant increase in the need for the implantation of a permanent pacemaker. This meta-study revealed no significant difference in permanent pacemaker implantation in the Cox-Maze group compared to the control group (mean: 8.0% vs. 4.7%; *P* = 0.128). However, Gillinov’s study showed there was a significantly higher rate of permanent pacemaker implantation in the Cox-Maze group than in the control group (21.5 vs. 8.1 implantations per 100 patient-years; 1 year incidence rate ratio, 2.64; 95% CI 1.20 to 6.41; *P* = 0.01). This relatively high rate may be attributable in part to the valve surgery itself in patients undergoing Cox-Maze rather than the Cox-Maze, which increases the risk of atrioventricular block.

In addition, institutional experience is of paramount importance due to the fact that a center might have higher morbidity or early postoperative mortality while introducing the Cox‐Maze technique. In Von Oppell’s study [23], they started using the monopolar pen to ensure a confluent ablation line between the left pulmonary veins and the mitral valve annulus and the tricuspid valve annulus without potentially injuring coronary arteries after the first three failure patients were treated only with the bipolar device, and could be interpreted as part of ‘learning curve’. Surgeons must know the specific characteristics of whatever device they plan to use to create transmural lesions and modify their technique accordingly. If surgeons are able to meet these criteria and increase the appropriate use of this effective surgical treatment, this would make a significant positive impact on patients with AF, not only by restoring sinus rhythm and reducing the risk of stroke but also by improving long-term [7]. Another criticism of port access surgery has been the prolonged CBP and ischemic times, which are associated with centers' experience. Our series is no exception, the reviewed data strongly suggest that CBP time seems to be prolonged with concomitant Cox‐Maze procedure. Therefore, a clear advantage of this technique remains valid for centers with substantial experience in antiarrhythmic surgery.

### Limitations

The present analysis has some limitations. Firstly, one thing to keep in mind is that our use of methods adapted from TSA applied to cumulative meta-analysis showed that the current evidence for Cox-Maze procedures maybe insufficient and inconclusive. Secondly, this study included all types of Cox-Maze surgeries, including Cox III, Cox IV, and some different additional surgical approaches, and the surgeon's individual experience at each center may have contributed to the bias, which highlights the need for future RCTs or large registries which evaluate maze ablation techniques according to subgroup. Thirdly, the current trial enrolled a substantial number of patients with “difficult to manage” atrial fibrillation, including elderly patients and patients with atrial fibrillation of relatively long duration before surgery. These factors have been associated with a reduced likelihood of ablation success. Fourthly, mitral valve replacement was higher than mitral valve repair in patients who underwent repeat mitral valve surgery, and we are not certain of the impact of these two subgroups on outcomes; subgroup analyses need to be performed by including a larger sample size of RCTs.

## Conclusions

The results of our meta‐analysis represent the first systematic evidence supporting the safety and efficacy of the Cox-Maze procedure in AF patients undergoing mitral valve surgery. No differences in terms of 12 months all-cause mortality, pacemaker implantation, stroke and thromboembolic events were observed between the two groups. Short and intermediate term SR outcomes follow-up was favorable, with 67.1% of patients receiving the combined Cox-Maze procedure being SR at 12 monthss in comparison to 21.4% in the control group. Whether these favorable results will continue during the long term remains to be seen; nevertheless, early results are encouraging. Thus, this meta-analysis indicates that surgical Cox-Maze can be performed in AF patients without increased risk of morbidity and mortality.

### Supplementary Information

**Additional file 1**. AF therapies preoperative.

**Additional file 2**. Antiarrhythmic drugs perioperative.

## Data Availability

The datasets generated and analyzed during the current study are available from the corresponding author on reasonable request.
