# Supplementary figures and images for: Safety and efficacy of Cox‐Maze procedure for atrial fibrillation during mitral valve surgery: a meta-analysis of randomized controlled trials
Source: J Cardiothorac Surg. 2024 Mar 19;19:140. doi: 10.1186/s13019-024-02622-0 (PMC10949564; doi:10.1186/s13019-024-02622-0)

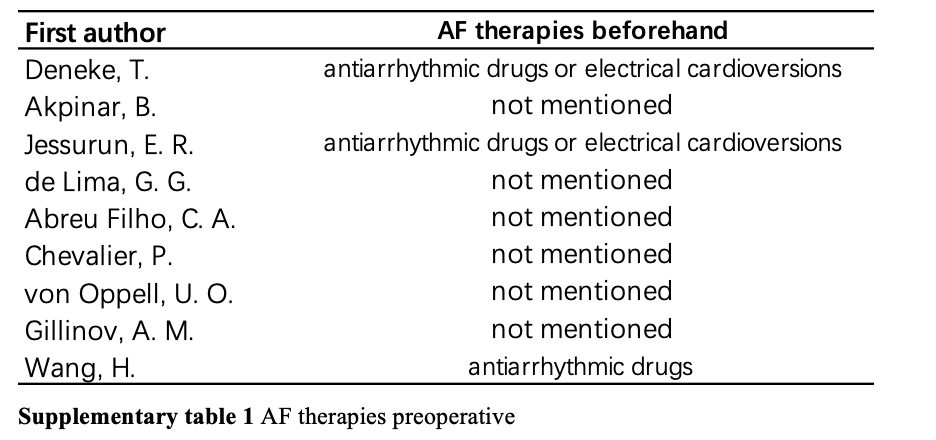

Supplement: Supplementary file 1 — Additional file 1. AF therapies preoperative. [file 13019_2024_2622_MOESM1_ESM.png]

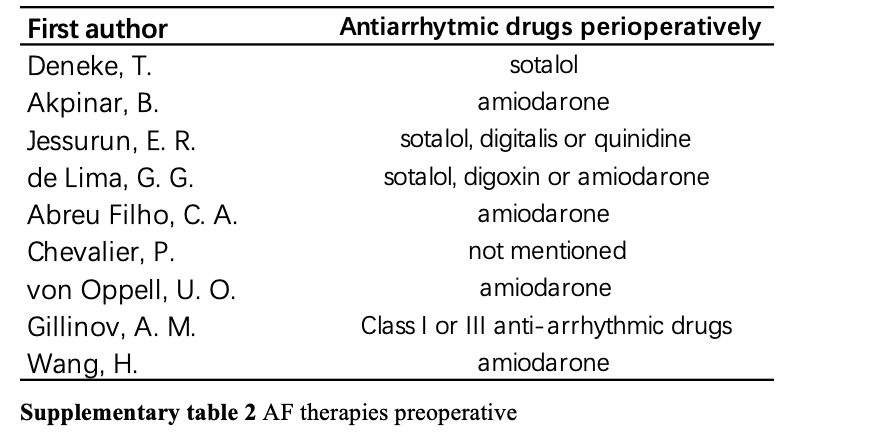

Supplement: Supplementary file 2 — Additional file 2. Antiarrhythmic drugs perioperative. [file 13019_2024_2622_MOESM2_ESM.png]
